# Rotating Machinery Fault Diagnosis Based on Improved Multiscale Amplitude-Aware Permutation Entropy and Multiclass Relevance Vector Machine

**DOI:** 10.3390/s19204542

**Published:** 2019-10-18

**Authors:** Yinsheng Chen, Tinghao Zhang, Wenjie Zhao, Zhongming Luo, Haijun Lin

**Affiliations:** 1 School of Measurement and Communication Engineering, Harbin University of Science and Technology, Harbin 150080, China; zhaowenjie@hrbust.edu.cn (W.Z.); lhjhlg@126.com (H.L.); 2 School of Electrical Engineering and Automation, Harbin Institute of Technology, Harbin 150080, China; zhangtinghaohit@163.com

**Keywords:** rotating machinery, fault diagnosis, fault severity, intrinsic time-scale decomposition, amplitude-aware permutation entropy, multiclass relevance vector machine

## Abstract

The health state of rotating machinery directly affects the overall performance of the mechanical system. The monitoring of the operation condition is very important to reduce the downtime and improve the production efficiency. This paper presents a novel rotating machinery fault diagnosis method based on the improved multiscale amplitude-aware permutation entropy (IMAAPE) and the multiclass relevance vector machine (mRVM) to provide the necessary information for maintenance decisions. Once the fault occurs, the vibration amplitude and frequency of rotating machinery obviously changes and therefore, the vibration signal contains a considerable amount of fault information. In order to effectively extract the fault features from the vibration signals, the intrinsic time-scale decomposition (ITD) was used to highlight the fault characteristics of the vibration signal by extracting the optimum proper rotation (PR) component. Subsequently, the IMAAPE was utilized to realize the fault feature extraction from the PR component. In the IMAAPE algorithm, the coarse-graining procedures in the multi-scale analysis were improved and the stability of fault feature extraction was promoted. The coarse-grained time series of vibration signals at different time scales were firstly obtained, and the sensitivity of the amplitude-aware permutation entropy (AAPE) to signal amplitude and frequency was adopted to realize the fault feature extraction of coarse-grained time series. The multi-classifier based on the mRVM was established by the fault feature set to identify the fault type and analyze the fault severity of rotating machinery. In order to demonstrate the effectiveness and feasibility of the proposed method, the experimental datasets of the rolling bearing and gearbox were used to verify the proposed fault diagnosis method respectively. The experimental results show that the proposed method can be applied to the fault type identification and the fault severity analysis of rotating machinery with high accuracy.

## 1. Introduction

Rotating machinery is one of the most common mechanical equipment, which plays an important role in industrial applications. It generally operates under tough working environments, which can eventually result in mechanical breakdown that lead to high maintenance costs, severe financial losses, and safety concerns [1,2]. As rotating machinery is the most malfunctioning part of a mechanical system, the fault diagnosis of rotating machinery has been a popular research topic in the industry. At present, there are many different theoretical methods to solve the fault diagnosis of rotating machinery, including a vibration signal analysis, acoustic emission, thermal imaging and multi-sensor fusion, etc [3]. In the condition monitoring and fault diagnosis technology of rotating machinery, the fault diagnosis technology based on vibration signals is widely used because of the close correlation between the vibration signal and mechanical structure [4,5].

The vibration signal is widely used in fault diagnosis of rotating machinery because it is easy to collect and monitor online. For example, when there is a local fault in the running process of the rolling bearing, each contact causes an instantaneous shock and stimulates the rolling bearing to conduct high-frequency free vibration attenuation according to its inherent frequency. The instantaneous impact caused by the failure has obvious periodicity, the impact frequency depends on the bearing speed, and the impact amplitude depends on the bearing fault size. Therefore, the impact characteristics caused by local damage should be extracted by signal analysis technology and then, fault identification should be conducted by the artificial classifier.

As rotating machinery works in the industrial environment, its vibration signal often contains the inherent vibration signal of rotating machinery, the fault impact signal and background noise. The vibration signals collected by the accelerometers have the characteristics of non-linearity, non-stationarity and impact [6]. Therefore, how to effectively extract fault signal characteristics from complex vibration signals and accurately identify these fault features are the key problems in the fault diagnosis of rotating machinery. Pattern recognition is one of the important methods to realize the vibration signal analysis of rotating machinery. Many scholars have made achievements in this field. Jiang et al. [7] decomposed the vibration signal by ensemble local characteristic-scale decomposition (ELCD) and obtained a series of intrinsic scale components (ISCs). The principle ISCs were selected and the permutation entropy (PE) values of these ISCs were calculated to construct the feature vector. Finally, the fault type of the rolling bearing is identified by the relevance vector machine (RVM) constructed by the feature vector set. Li et al. [8] proposed a rolling bearing fault diagnosis method based on multiscale permutation entropy (MPE) and improved the support vector machine based on the binary tree (ISVM-BT). Local mean decomposition (LMD) was utilized to decompose the vibration signal of the rolling bearing into a set of product functions (PFs), and the MPE extracted the fault features of the rolling bearing from PFs. The ISVM-BT established by a feature set effectively identified the fault type automatically. Chen et al. [9] presented an integrated fault diagnosis method for a gearbox using complementary ensemble empirical mode decomposition (CEEMD), sample entropy (SampEn) and a probabilistic neural network (PNN). CEEMD decomposed the vibration signal of the gearbox into a set of intrinsic mode functions (IMFs). The fault features were extracted by SampEn from each IMF. Then, the PNN was used as the classifier to identify the fault type of the gearbox.

Due to the nonlinearity and non-stationarity of the vibration signals of rotating machinery a time-frequency analysis method is often used to solve the problem of feature extraction of the vibration signals of rotating machinery. The fast Fourier transform (FFT) is a classical time-frequency analysis method, but it is only suitable for solving the problem of stationary signal analysis. The wavelet transform (WT) is also a classical time-frequency analysis method that can preset the time and frequency window of interest. However, the WT is not an adaptive signal decomposition method, and it requires the kernel function and its parameters to be set in advance. The wavelet packet transform (WPT) can select the frequency resolution and the WPT is more flexible than the WT. However, the WPT is still not an adaptive time-frequency analysis method. The empirical mode decomposition (EMD) is a self-adaptive time-frequency method that can adaptively decompose the vibration signal into a set of intrinsic mode functions (IMFs) that contain the amplitude and frequency characteristics. However, the EMD has the end effect and mode mixing problem in that the stability of the IMFs is poor, which affects the subsequent feature extraction process. In order to solve the problems existing in EMD, EEMD and a complete ensemble empirical mode decomposition (CEEMD) are proposed [6].

In recent years, due to the fact that fault information contained in the vibration signals can be extracted more effectively at different time scales, a large number of scholars have applied a multiscale entropy (MSE) algorithm and its variants to fault feature extraction of rotating machinery [10,11]. In addition to the MSE algorithm [12], some scholars proposed many fault feature extraction methods of the vibration signal based on the multiscale permutation entropy (MPE) [13,14,15] and multiscale fuzzy entropy (MFE) [16,17]. However, the commonly used entropy theoretical methods still have some limitations. The poor stability of the approximate entropy (ApEn) results leads to its excessive dependence on the length of the time series. The calculation efficiency of the sample entropy (SampEn) is low, which is not suitable for analyzing long time series. When calculating the PE value of the time series, the PE algorithm does not consider the average amplitude of the time series. The different signals with significantly different mean amplitudes may be counted in the same order. Moreover, if there are elements with the same amplitude in the time series, the results calculated by the PE will be random. At present, the performance of the fault feature extraction method based on entropy theory needs to be improved. In order to solve the above problems, the amplitude-aware permutation entropy (AAPE) was proposed by Azami and Escudero [18] to improve the classical PE. The AAPE is sensitive to the changes in the amplitude, in addition to the frequency that can highlight the fault information contained in the vibration signal more effectively than the PE. In the coarse-graining procedures of the MSE and MPE, the length of the coarse-grained time series decreases with the increase of the scale factor. Therefore, when the scale factor is large, the entropy value of the coarse-grained time series is unstable. The literature [19] improved the coarse-graining procedure of MSE by making the computation stable and reliable in the case of a large time scale through the sliding averaging process, so as to solve the shortcomings of the traditional MSE.

After the fault features are extracted from the vibration signals of rotating machinery, a high performance classifier is needed to identify the fault types and fault severity. Many artificial intelligence techniques have been adopted to realize the fault diagnosis of rotating machinery, such as the artificial neural network (ANN) [20], support vector machine (SVM) [21], random forest (RF) [22]. The structure of the ANN is usually set by experience and its recognition rate is related to the number of training samples. Although the SVM can realize classification with high accuracy under the dichotomist condition of small training samples, the SVM requires multiple dichotomers to realize multiple classifications and further, the selection of the kernel function directly affects the classification accuracy. It is difficult for the random forest classifier to obtain the most ideal parameters, and the selection of parameters has a great impact on the recognition results. The relevance vector machine (RVM) [7] is more sparse than the SVM, more suitable for online monitoring, and its generalization ability is better than the SVM. However, like the SVM, the RVM is a binary classifier and cannot directly implement multiple classifications. The multiclass relevance vector machine (mRVM) [23] is an extended algorithm that can directly classify the input samples into multiple categories and output the probabilities belonging to each category. The unique properties of the mRVM are suitable for the multi-fault identification of rotating machinery fault diagnosis [6,24].

In view of the above problems in fault diagnosis of rotating machinery based on the pattern recognition method, this paper presents a novel fault diagnosis method based on the improved multiscale amplitude-aware permutation entropy (IMAAPE) and the mRVM for rotating machinery. The main contributions of this paper are summarized as follows:

(1) As the AAPE is very sensitive to the amplitude change of the vibration signal, the vibration of rotating machinery needs to be pre-processed before the feature extraction to minimize the interference of external noise to the vibration signal. The intrinsic time-scale decomposition (ITD) was used to decompose the vibration signal of rotating machinery into a group of proper rotation components stably, among which the optimum PR component can highlight the main time-frequency characteristics of the vibration signal so as to facilitate the subsequent fault feature extraction.

(2) The performance of the AAPE improved. A fault feature extraction method of rotating machinery based on the IMAAPE is proposed for the first time. The IMAAPE improves the coarse-graining procedure in a multiscale analysis and adopts the characteristics of the AAPE sensitive to the amplitude and frequency changes of the vibration signal. The IMAAPE can calculate the AAPE values in different time scales and construct the feature vectors, which can effectively describe the fault features contained in the vibration signals of rotating machinery.

(3) The mRVM multi-classifier is trained to realize fault identification and fault severity analysis of rotating machinery. In this paper, two different realization methods of the mRVM and the effect of parameter selection on the identification accuracy of rotating machinery fault types are discussed by comparing experiments.

The organization of the rest of this paper is as follows. Section 2 introduces the theoretical basis of the methodologies adopted in this paper. The proposed fault diagnosis method is described in Section 3. Section 4 verifies the feasibility and effectiveness of the proposed fault diagnosis method by rolling bearing experiments and gearbox experiments, respectively. Finally, conclusions are drawn in Section 5.

## 2. Methodologies

### 2.1. Instrinsic Time-Scale Decomposition

The ITD is an algorithm for the efficient and precise time-frequency-energy (TFE) analysis of signals. The ITD can decompose a complex time series into a series of proper rotation (PR) components and accurately extract the intrinsic instantaneous amplitude, frequency information and other morphological characteristics of the complex time series, which is suitable for the analysis of non-stationary and nonlinear signals [25].

Let $X_{t}$ be the complex time series to be analyzed and define ℒ as the baseline extraction factor. ℒ can extract the baseline signal $L_{t}=ℒX_{t}$ from $X_{t}$, then $X_{t}$ can be decomposed into:

(1) $$X_{t}=ℒX_{t}+(1-ℒ)X_{t}=L_{t}+H_{t}$$

where $L_{t}$ is the baseline signal, and $H_{t}$ is the PR component.

The main steps of the ITD algorithm are as follows:

Assuming that $\left\{\tau_{k},k=1,2,\ldots \right\}$ represents the local extrema of signal $X_{t}$, the default $\tau_{0}=0$. $L_{t}$ and $H_{t}$ are defined in the interval $\left[0,\tau_{k}\right]$, and $X_{t}$ is valid in the interval $t\in \left[0,\tau_{k+2}\right]$. In the successive extrema interval $\left(\tau_{k},\tau_{k+1}\right]$, the extracted baseline signal $L_{t}$ is expressed as:

(2) $$ℒX_{t}=L_{t}=L_{k}+\left(\frac{L_{k+1}-L_{k}}{X_{k+1}-X_{k}}\right)\left(X_{t}-X_{k}\right),t\in (\tau_{k},\tau_{k+1}]$$

in which:

(3) $$L_{k+1}=\alpha \left[X_{k}+\left(\frac{\tau_{k+1}-\tau_{k}}{\tau_{k+2}-\tau_{k}}\right)\left(X_{k+2}-X_{k}\right)\right]+(1-\alpha )X_{k+1}$$

where α is a linear scaling factor used to adjust the amplitude of the extracted PR component, α∈[0,1] [25].

According to Equation (2) and Equation (3), the PR component $H_{t}$ can be expressed as:

(4) $$ℋX_{t}\equiv (1-ℒ)X_{t}=H_{t}=X_{t}-L_{t}$$

where ℋ is proper rotation extraction operator.

The baseline signal $L_{t}$ can be taken as the input signal of the next decomposition and the above steps can be repeated to obtain a series of PR components. The termination condition of decomposition is that the baseline signal $L_{t}$ becomes monotonous or less than a certain preset value.

After the ITD, the time series $X_{t}$ is decomposed into a series of PR components and a monotone trend component. The kurtosis value of the signal can effectively describe the pulse characteristic of the signal. The higher the kurtosis value, the richer the impact features contained in the signal. Therefore, the PR component with the maximum kurtosis value is defined as the optimum PR component and its calculation process is expressed as follows:

(5) $$K_{i}=\frac{1}{n}\sum_{k=1}^{n}PR_{ik}^{4}$$

(6) $$U_{i}=\frac{K_{i}}{\sum_{i=1}^{m}K_{i}}$$

where $K_{i}$ represents the kurtosis value of the *i*th PR component and n represents the length of the time series. $U_{i}$ is the normalized kurtosis value of the *i*th PR component and m is the number of PR components. The optimum proper rotation component selects the PR component corresponding to the maximum value of $U_{i}$.

### 2.2. Multiscale Entropy

The entropy analysis of the time series from a single scale may lose some important information of the original signal. The multiscale entropy (MSE) was proposed by Costa M. to represent the complexity of a signal. The MSE relies on the computation of the sample entropy over a range of scales to extract the characteristic information of the complex signal in different time scales [26]. The MSE algorithm is composed of two steps:

(1) The coarse-graining procedure derives a set of time series representing the system dynamics on different time scales. The coarse-graining procedure for scale i is obtained by averaging the samples of the time series inside the consecutive but non overlapping windows of length i. For a monovariate discrete signal of $\left\{X_{i}\}=\{x_{1},x_{2},\ldots ,x_{N}\right\}$, the coarse-grained time series can be computed as:

(7) $$y_{j}^{\tau }=\frac{1}{\tau }\sum_{i=(j-1)\tau +1}^{j\tau }x_{i},1\leq j\leq \frac{N}{\tau }$$

where $y_{j}^{\tau }$ represents the new-time series obtained after the coarse-graining procedure when the scale factor is τ. The length of the coarse-grained time series $\left\{y_{}^{\tau }\right\}$ is N/τ.

(2) Then, the sample entropy of each coarse-grained time series is calculated and n sample entropy values of different time scales are obtained to describe the signal characteristics of the original time series.

### 2.3. Amplitude-Aware Permutation Entropy

Bandt put forward the concept of basic permutation entropy in 2002 [27]. At present, the PE is widely used in the analysis of complex time series signals to measure the complexity of a nonlinear and non-stationary signal. The calculation process of the PE is as follows:

Assume the given time series $x=\{x_{1},x_{2},\ldots ,x_{N}\}$ with length N, and for each time point t, embed the signal x in a *d*-dimensional space to obtain the reconstruction vectors $X_{t}^{d,l}=\{x_{t},x_{t+l},\ldots ,x_{t+(d-2)l},x_{t+(d-1)l}\},t=1,2,\ldots ,N-(d-1)l$, where d and l denote the embedding dimension and the time delay, respectively. Each vector $X_{t}^{d,l}$ is arranged in an increasing order as $\left\{x_{t+(j_{1}-1)l},x_{t+(j_{2}-1)l},\ldots ,x_{t+(j_{d-1}-2)l},x_{t+(j_{d}-1)l}\right\}$, where $j_{*}$ is the index of the element in the reconstruction vector. Therefore, when the embedding dimension is d, there are d! potential ordinal pattern and the *i*th permutation is called as $\pi_{i}$. For each $\pi_{i}$, $p(\pi_{i})$ represents the occurrence probability as follows:

(8) $$p(\pi_{i})=\frac{f(\pi_{i})}{N-d+1}$$

where $f(\pi_{i})$ is the function that counts the number of occurrences of $\pi_{i}$. Whenever the inner elements of $X_{t}^{d,l}$ are arranged in order of $\pi_{i}$, $f(\pi_{i})$ increases by 1. The definition of the PE is as follows:

(9) $$PE(x,d,l)=-\sum_{\pi_{i}=1}^{\pi_{i}=d!}p(\pi_{i})\ln p(\pi_{i})$$

**Figure 1:**
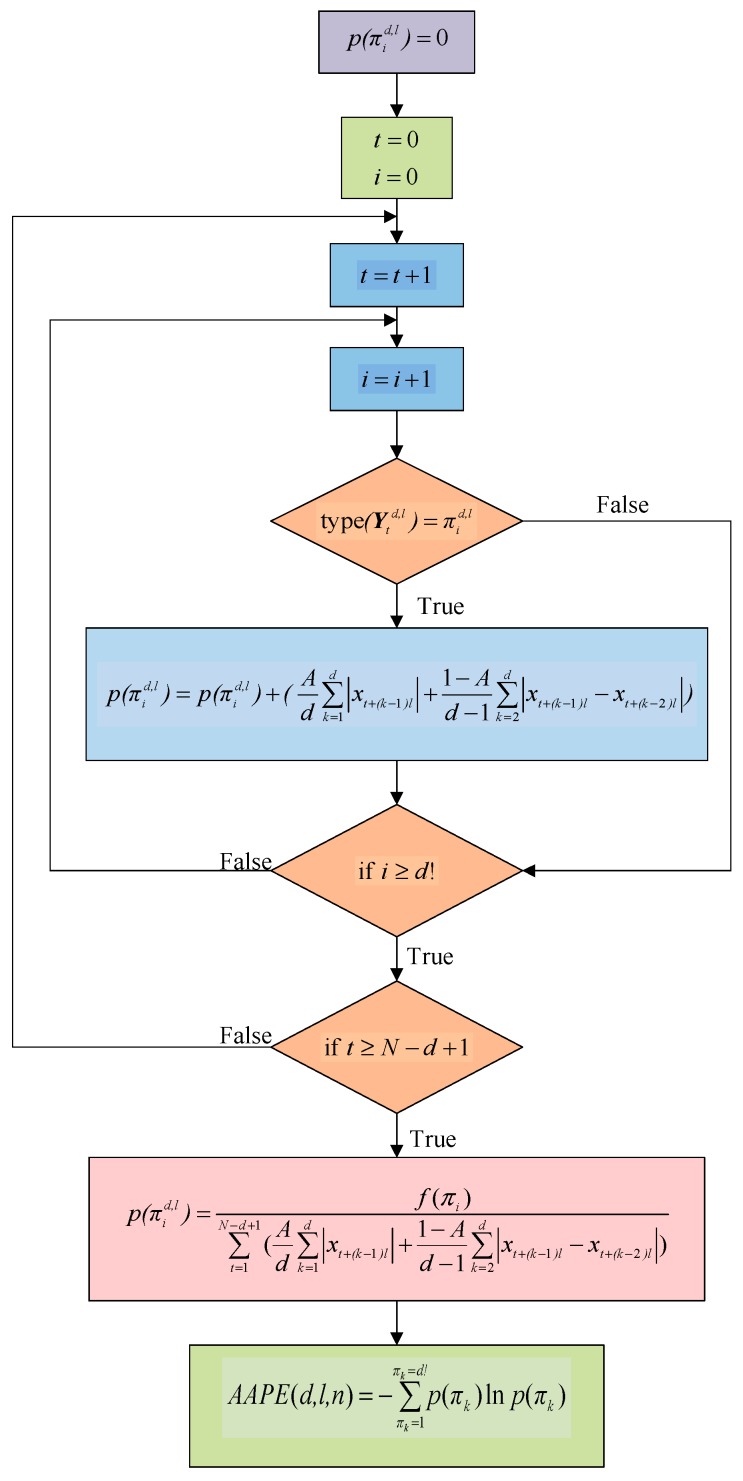
The flow chart of the amplitude-aware permutation entropy (AAPE) algorithm.

However, there are two main problems in describing the complex time series by the PE. First, the traditional PE only considers the ordinal structure of a time series, but ignores the amplitude information of the corresponding elements in the time series. Second, the effect of the elements with equal amplitude on the PE value in the time series is not clearly explained. In view of these, Azami and Escudero proposed the amplitude-aware permutation entropy (AAPE) to improve the sensitivity of the PE to the amplitude and frequency of the time series. The flow chart of the AAPE algorithm is shown in Figure 1 [18].

Assuming that the initial value of $p(\pi_{i}^{d,l})$ is 0, for the time series $X_{t}^{d,l}$, when t gradually increases from 1 to N−d+1, $p(\pi_{i}^{d,l})$ should be updated whenever $\pi_{i}^{d,l}$ appears.

(10) $$p(\pi_{i}^{d,l})=p(\pi_{i}^{d,l})+\left(\frac{A}{d}\sum_{k=1}^{d}\left|x_{t+(k-1)l}\right|+\frac{1-A}{d-1}\sum_{k=2}^{d}\left|x_{t+(k-1)l}-x_{t+(k-2)l}\right|\right)$$

where A∈[0,1] is the adjustment coefficient to adjust the weight of the signal amplitude mean and the deviation between the amplitudes. Therefore, the probability of $p(\pi_{i}^{d,l})$ appearing in the whole time series is $\pi_{i}^{d,l}$.

(11) $$\frac{p(\pi_{i}^{d,l})}{\sum_{t=1}^{N-d+1}\left(\frac{A}{d}\sum_{k=1}^{d}\left|x_{t+(k-1)l}\right|+\frac{1-A}{d-1}\sum_{k=2}^{d}\left|x_{t+(k-1)l}-x_{t+(k-2)l}\right|\right)}$$

The AAPE calculation of the time series can be expressed as follows:

(12) $$AAPE(d,l,n)=-\sum_{\pi_{k}=1}^{\pi_{k}=d!}p(\pi_{k})\ln p(\pi_{k})$$

### 2.4. Multiclass Relevance Vector Machine

The traditional RVM is a binary classifier which cannot directly solve the multi-classification problem. The multiclass relevance vector machine (mRVM) effectively solves the multi-classification application problem of the traditional RVM. The basic principle of the mRVM is described below.

The input training data sample set is denoted as $T={\left\{x_{i},t_{i}\right\}}_{i=1}^{N}$, where $x_{i}\in R^{D}$ is D dimensional input vector and t∈{1,2,⋯,C} is the corresponding category tag. The kernel function is set as $K\in R^{N\times N}$, the auxiliary variable $Y\in R^{C\times N}$ is introduced as the target of weight parameter $w^{T}K$, and obtain:

(13) $$y_{cn}|w_{c},k_{n}\sim N_{ycn}\left(w_{c}{}^{T}k_{n},1\right)$$

The continuous nature of Y allows, not only multiple class discrimination by the multinomial probit link $t_{n}=i\;\mathrm{if}\;y_{ni}>y_{nj}\;\forall j\neq i$, but also a probabilistic output for class membership via the multinomial probit likelihood function,

(14) $$P(t_{n}=i|w,k_{n})=\varepsilon_{p(u)}\left\{\prod_{j\neq i}\Phi (u+{\left(w_{i}-w_{j}\right)}^{Τ}k_{n})\right\}$$

where ε is the expectation of the standard normal distribution p(u)~N(0,1) and Φ is the Gaussian cumulative distribution function.

In order to ensure the sparsity of the mRVM, similar to the RVM, a normal prior distribution with mean value of 0 and variance of $\alpha_{nc}^{-1}$ is introduced for weight parameter w. $\alpha_{nc}^{}$ belongs to the prior parameter matrix $A\in R^{N\times C}$ and obeys the Gamma distribution of parameters τ,υ. $\tau ,\upsilon (<10^{-5})$ can guarantee the sparsity of mRVM.

(15) $$P(w|Y)\propto P\left(Y|w\right)P\left(w|A\right)\propto \prod_{c=1}^{C}N{\left({\left(KK^{T}+A_{c}\right)}^{-1}Ky_{c}^{Τ},\left(KK^{Τ}+A_{c}\right)\right)}^{-1}$$

**Figure 2:**
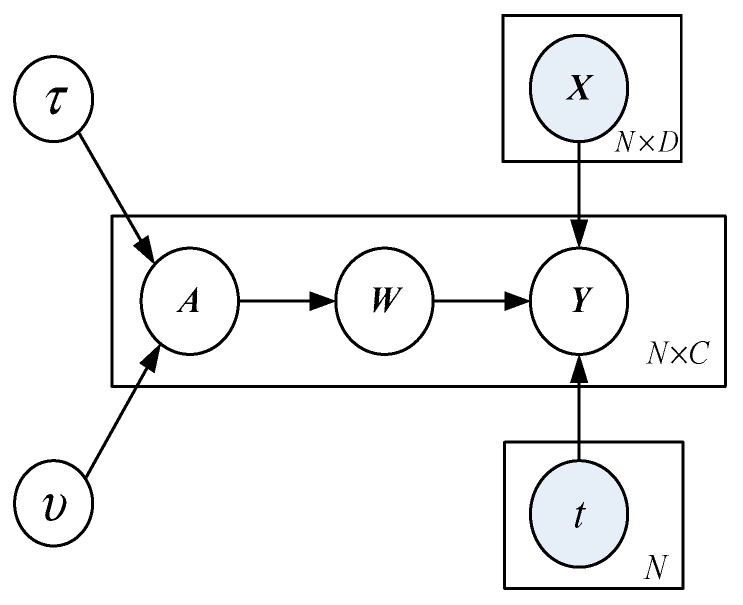
Plates diagram of the multiclass relevance vector machine (mRVM) model.

The regressors w closed-form posterior can be derived based on Figure 2. In Equation (15), $A_{c}$ is a diagonal matrix derived from the c column of A which expresses the scales $\alpha_{ic}^{}$ across samples. Then, through the maximum posterior probability estimation, the equation can be obtained as:

(16) $$\hat{w}=\underset{w}{\arg\max}P(w|Y,A,K)$$

When category i is given, the update method based on weight parameters is as follows:

(17) $${\hat{w}}_{c}={\left(KK^{Τ}+A_{c}\right)}^{-1}Ky_{c}^{Τ}$$

For a certain category, the posterior expectation of auxiliary variables is:

(18) $${\tilde{y}}_{in}={\hat{w}}_{i}^{T}k_{n}-(\sum_{j\neq i}{\tilde{y}}_{in}-{\hat{w}}_{i}{}^{T}k_{n})$$

For ∀c≠i,

(19) $${\tilde{y}}_{cn}\leftarrow {\hat{w}}_{c}^{T}k_{n}-\frac{\varepsilon_{p(u)}\left\{N_{u}({\hat{w}}_{c}^{T}k_{n}-{\hat{w}}_{i}^{T}k_{n},1)\Phi_{u}^{n,i,c}\right\}}{\varepsilon_{p(u)}\left\{\Phi (u+{\hat{w}}_{i}^{T}k_{n}-{\hat{w}}_{c}^{T}k_{n})\Phi_{u}^{n,i,c}\right\}}$$

and for the ith class:

(20) $${\tilde{y}}_{in}\leftarrow {\hat{w}}_{i}^{T}k_{n}-\left(\sum_{j\neq i}{\tilde{y}}_{jn}-{\hat{w}}_{j}^{T}k_{n}\right)$$

where the “tilde” symbol above y denotes the expected value and Φ is a normalized cumulative distribution function, $\Phi_{u}^{n,i,c}=\prod_{j\neq i,c}\Phi (u+{\hat{w}}_{i}^{T}k_{n}-{\hat{w}}_{j}^{T}k_{n})$.

The posterior probability distribution of the prior parameters of the weight vector is:

(21) $$P(A|w)\propto P(w|A)P(A|\tau ,\upsilon )\propto \prod_{c=1}^{C}\prod_{n=1}^{N}G(\tau +\frac{1}{2},\frac{w_{nc}^{2}+2\upsilon }{2})$$

Psorakis proposed two training methods of the mRVM in the literature [23] and the difference between them lies in the different nuclear operation modes at the training stage. The mRVM_1_ follows the construction process, starting with an empty sample set, gradually adding samples according to their contribution to the method, or deleting samples with a low contribution to the method. The mRVM_1_ has two convergence principles: conv_1_ and conv_2_. The mRVM_1__conv_1_ follows the principle described by [28]. The mRVM_1__conv_2_ adds the limit of the minimum number of iterations to the mRVM_1__conv_1_. The mRVM_2_ follows a top-down process, first loading the entire training sample set and then removing the unnecessary samples during the training process. The mRVM_2_ has two convergence principles: conv_A_ and conv_N_. For the mRVM_2__conv_A_ principle, $\left|\log A^{\left(k\right)}-\log A^{\left(k-1\right)}\right|<\varsigma$ indicates the iterative convergence. For the mRVM_2__conv_N_, the number of iterations is limited to $\lambda N_{train}$.

## 3. The Proposed Fault Diagnosis Method

The main process of the proposed fault diagnosis method of rotating machinery includes signal preprocessing, fault feature extraction and fault identification. The principle of the fault diagnosis method proposed in this paper is introduced below.

### 3.1. Signal Preprocessing

Due to the fault of rotating machinery, the vibration signal has impact characteristics and the impact amplitudes are obviously different with different fault severity. In order to reduce the influence of external interference on the vibration signals and highlight the fault features of the vibration signals, it is necessary to preprocess the vibration signals before the feature extraction.

Although different from the time-frequency analysis method such as the EMD, EEMD and LMD, the ITD is used to highlight the major amplitude variations in the vibration signals. The ITD algorithm is adopted to decompose the vibration signal into a sum of proper rotation components, for which instantaneous frequency and amplitude, as well as a monotonic trend, are well defined. The ITD can effectively suppress the mode mixing and end effect. The optimum proper rotation component is selected for further fault feature extraction because it contains the most obvious fault features. The calculation process of signal preprocessing based on the ITD can be referred to Section 2.1.

### 3.2. Feature Extraction

In order to effectively extract fault features of the vibration signals, an improved multi-scale amplitude-aware permutation entropy (IMAAPE) algorithm is proposed in this paper. This method improves the coarse-graining procedures in a multi-scale analysis and improves the stability of the fault feature extraction. In the classical MSE algorithm, when the scale factor τ is high, the number of elements in the coarse-grained time series decreases, which leads to instability of the entropy measure. In order to solve the problem of shortening the length of the time series after the MSE coarse-graining procedure, relevant scholars have improved the coarse-graining procedure [19].

**Figure 3:**
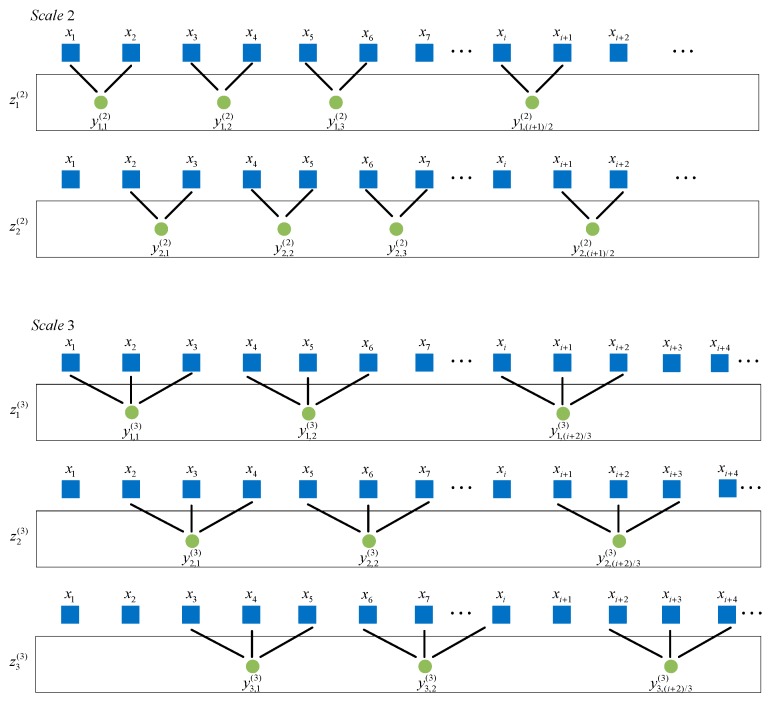
The improved coarse-graining procedures for scale factor τ = 2 and τ = 3.

Supposing that the time series to be analyzed is $\left\{x_{1},x_{2},\ldots ,x_{N}\right\}$, a set of coarse-grained time series $z_{i}^{\left(\tau \right)}=\left\{y_{i,1}^{\left(\tau \right)},y_{i,2}^{\left(\tau \right)},\cdots \right\}$ is generated by the improved coarse-graining procedure where $y_{i,j}^{\left(\tau \right)}=\frac{\sum_{f=0}^{\tau -1}x_{f+i+\tau (i-1)}}{\tau }$, τ=1,2,…,n. The improved coarse-graining procedures for scale factor τ=2 and τ=3 are shown in Figure 3.

For each scale factor τ and embedded dimension d, the AAPE value of each time series in $z_{i}^{\left(\tau \right)}|(i=1,2,\cdots ,\tau )$ is calculated respectively, and its average value is defined as IMAAPE,

(22) $$IMAAPE(x,\tau ,d)=\frac{1}{\tau }\sum_{i=1}^{\tau }AAPE(z_{i}^{\left(\tau \right)})$$

### 3.3. Fault Identification

The high-performance multi-classifier can realize the fault type identification and further, a fault severity analysis of rotating machinery. The mRVM is adopted to analyze and identify the fault features of rotating machinery in this paper. After the feature extraction of the vibration signal samples with different fault types and fault severity by IMAAPE, a fault feature set is formed to model the mRVM classifier. The established mRVM classifier can identify the fault type and analyze the fault severity of rotating machinery by extracting the IMAAPE fault feature from the vibration signals.

### 3.4. Fault Diagnosis Procedure

**Figure 4:**
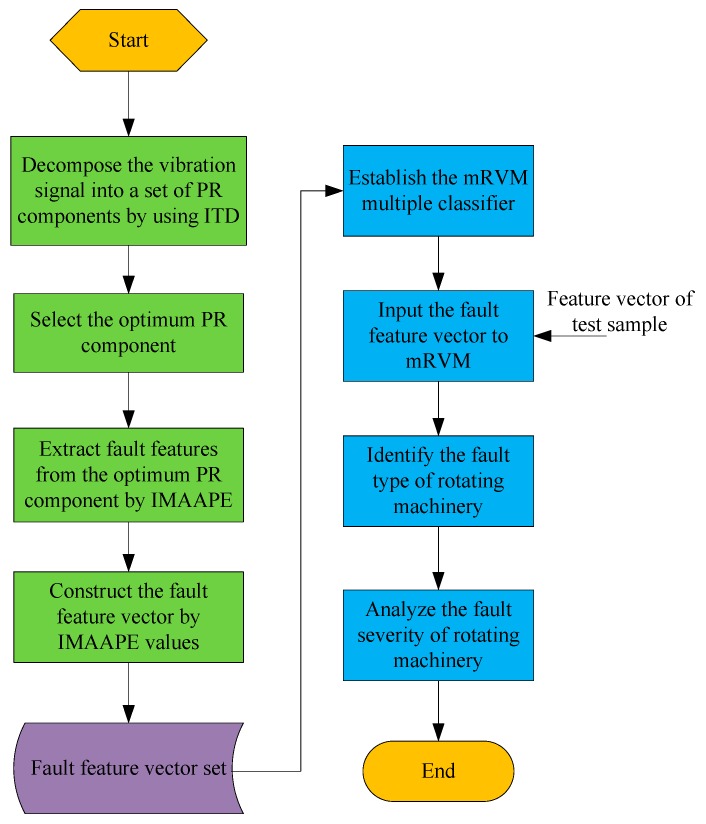
The proposed fault diagnosis procedure based on the improved multiscale amplitude-aware permutation entropy (IMAAPE) and mRVM.

The fault diagnosis procedure of rotating machinery proposed in this paper is shown in Figure 4. The whole procedure of the fault diagnosis method consists of two parts: the training part and testing part. In the training process, the vibration signals of rotating machinery under different fault states are collected according to a fixed sampling frequency to form the vibration signal sample set. The ITD is used to decompose the vibration signal into a set of PR components and the optimum PR component is selected to highlight the fault characteristics of rotating machinery. Then, the IMAAPE algorithm proposed in this paper is used to extract the features of the optimum PR component and construct the feature vector to accurately describe the fault type and fault severity. The fault feature vector set is constructed by all IMAAPE feature vectors that are extracted from the vibration signals in the vibration signal sample set. The mRVM multiple classifier is established by the fault feature vector set. In the testing process, the vibration signals of rotating machinery are collected by vibration accelerometers in real time. The fault features contained in the vibration signals are effectively extracted by the feature extraction method based on the IMAAPE proposed in this paper. Finally, the fault type and fault severity of rotating machinery are estimated by the mRVM classifier.

## 4. Experiment and Analysis

In order to verify the feasibility and effectiveness of the fault diagnosis method of rotating machinery proposed in this paper, the rolling bearing and gearbox are taken as examples to carry out the experiments and analysis. The rolling bearing experiment adopts the famous public data set provided by Case Western Reserve University Bearing Data Center [29]. The gearbox experiment is carried out at the QPZZ-II vibration analysis and fault diagnosis test platform system of rotating machinery manufactured by Jiangsu Qianpeng Diagnosis Engineering Co., Ltd. (Zhenjiang, China) [30].

### 4.1. Fault Diagnosis Experiment of Rolling Bearing

#### 4.1.1. Experimental Platform and Data Set

**Figure 5:**
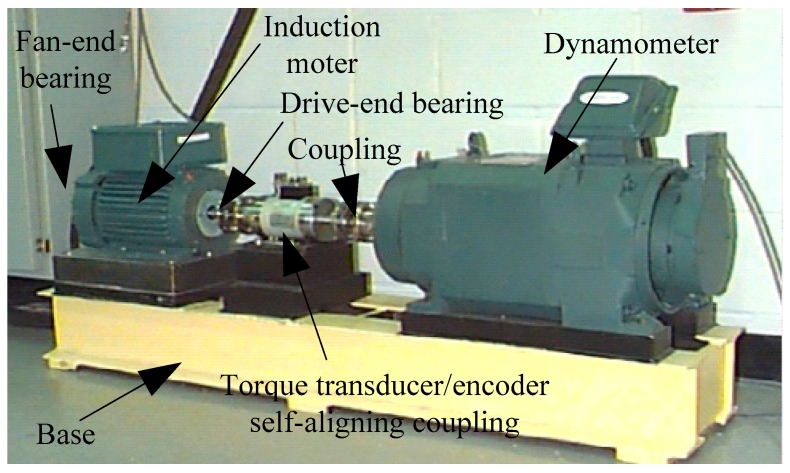
The experimental platform of rolling bearing from Bearing Data Center of Case Western Reserve University.

**Figure 6:**
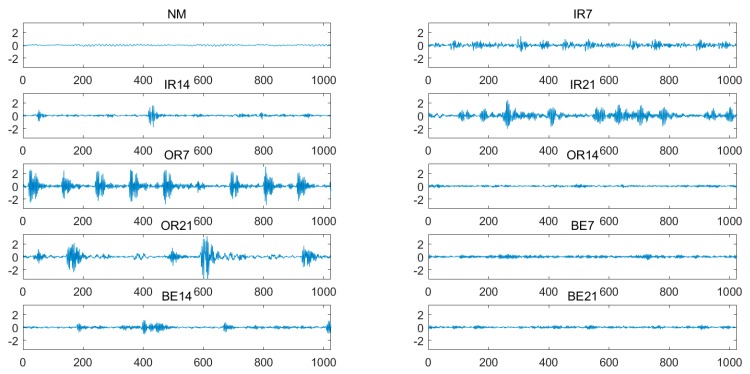
The vibration signal waveforms of rolling bearing in different fault states at load 0 hp.

The experimental platform designed by Case Western Reserve University Bearing Data Center is shown in Figure 5. The vibration data was collected using accelerometers, which were attached to the housing with magnetic bases. The accelerometers were placed at the 12 o’clock position at both the drive end and fan end of the motor housing. During some experiments, an accelerometer was attached to the motor supporting base plate as well. The vibration signals were collected using a 16 channel DAT recorder. In this paper, normal (Norm), inner race (IR) fault, outer race (OR) fault and ball elements (BE) fault are used for the experiments. The experimental sample description and experimental sample distribution under different load conditions of the rolling bearing are shown in Table 1 and Table 2, respectively. The vibration signal waveforms of the rolling bearing in different fault types with different fault severity at load 0 hp (1 hp = 746 w) are shown in Figure 6. As shown in Figure 6, the amplitude and frequency of the vibration signals of the rolling bearing under different fault states and fault severity are different.

#### 4.1.2. Fault Feature Extraction

**Figure 7:**
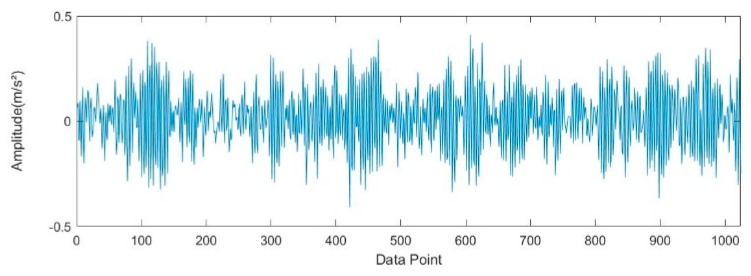
The vibration signal waveform of rolling bearing with the ball elements fault under the fault diameter of 7mils with the load 0 hp.

**Figure 8:**
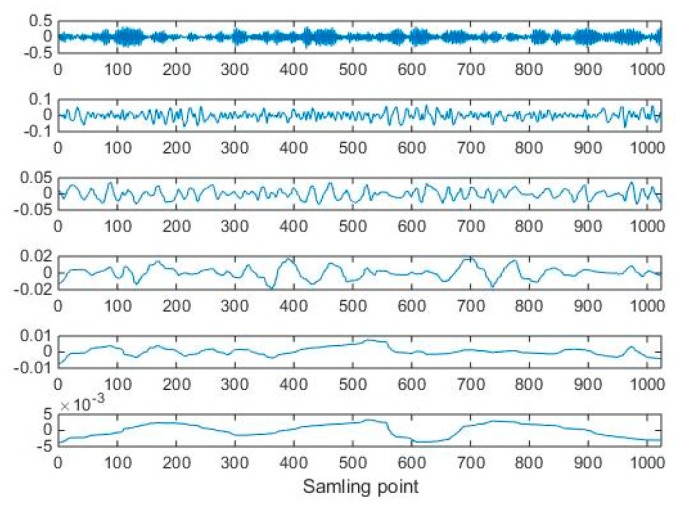
The intrinsic time-scale decomposition (ITD) results of the vibration signal with the ball elements of the rolling bearing under the fault diameter of 7 mils with the load 0 hp.

A vibration signal waveform of the rolling bearing with ball elements fault under the fault diameter of 7 mils with the load 0 hp is shown in Figure 7. The vibration signal is decomposed by the ITD and the ITD decomposition results (PR components) are shown in Figure 8. The PR component with the largest amplitude is the optimum PR component as shown in Figure 8. The optimum PR component highlights the frequency and amplitude characteristics of the ball elements fault.

**Figure 9:**
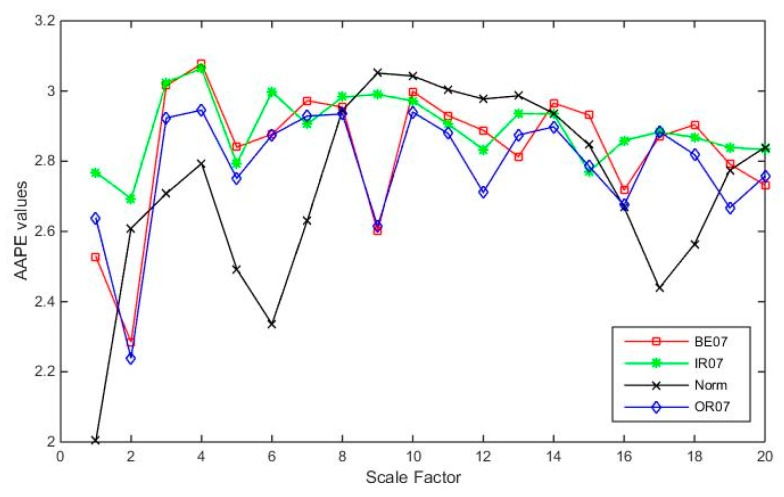
IMAAPE feature vectors for different fault types under the fault diameter of 7 mils with the load 0 hp.

**Figure 10:**
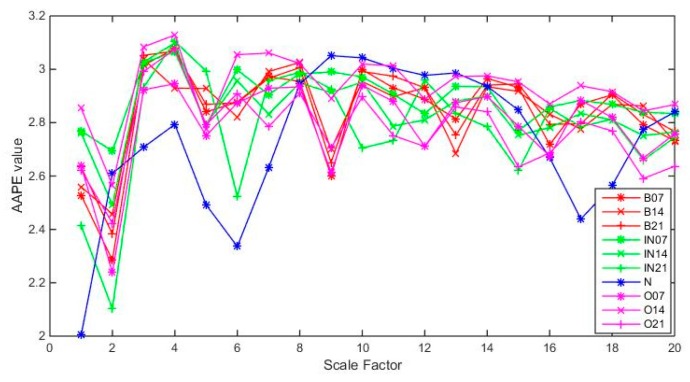
IMAAPE feature vectors for different fault types under different fault diameters with the load 0 hp.

Figure 9 shows the IMAAPE feature vectors for different fault types under the fault diameter of 7 mils with load 0 hp. Figure 10 shows the IMAAPE feature vectors for different fault types under the fault diameter of 7 mils with the load 0 hp. The IMAAPE can highlight the fault characteristics under different scale factors. It can be seen that the feature vectors of different fault types with different fault severity of the rolling bearings have certain differences. It is the difference of the feature vectors in different fault states that makes it possible to identify the fault types and analyze the fault severity.

**Figure 11:**
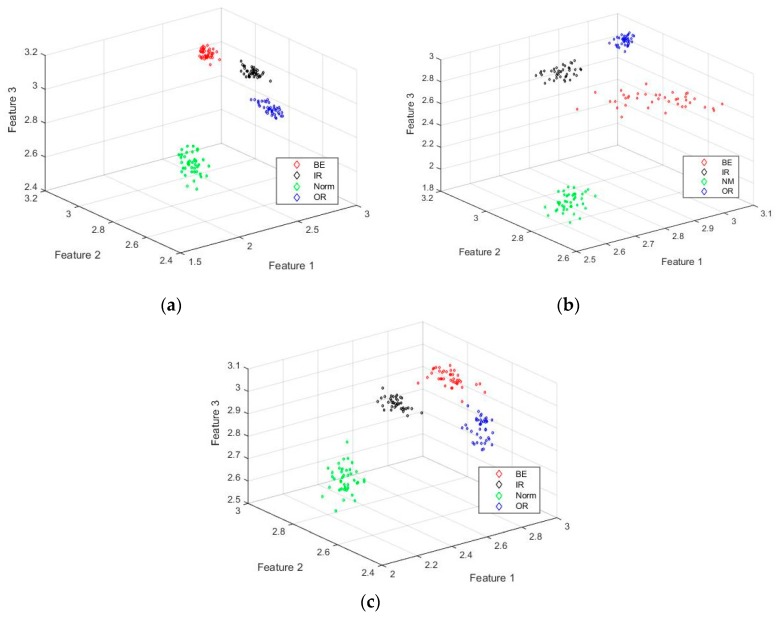
Three dimensions of the IMAAPE feature vectors for different fault types under different fault diameters with the load 0 hp. (**a**) fault diameter is 7 mils; (**b**) fault diameter is 14 mils; (**c**) fault diameter is 21 mils.

**Figure 12:**
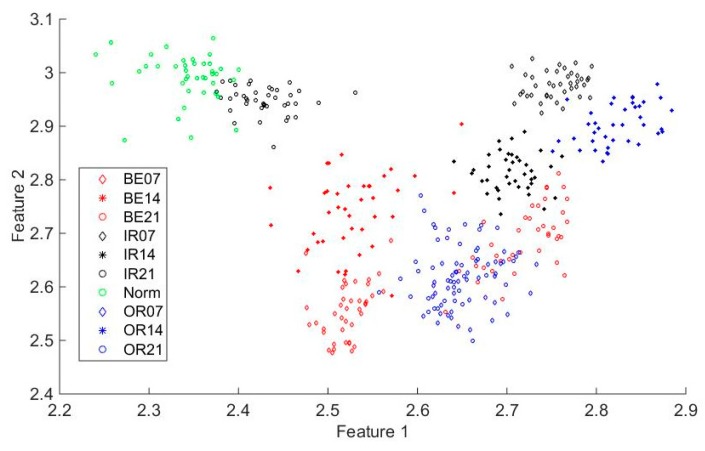
Two dimensions of the IMAAPE feature vectors for different fault types under different fault diameters with the load 0 hp.

The three dimensions of the IMAAPE feature vectors for different fault types under different fault diameters with load 0 hp is shown in Figure 11. It can be seen from the Figure 11 that the IMAAPE fault feature extraction method can make feature vectors extracted under different fault types have good clustering characteristics under different fault diameter conditions. Figure 12 shows the two dimensions of the IMAAPE feature vectors for different fault types under different fault diameters with the load 0 hp. It can be seen from Figure 12 that, although the fault features have different fault severity, the IMAAPE feature extraction method can also have good separability. The IMAAPE fault feature extraction method can provide an effective means for the fault severity analysis of the rolling bearings. The between-class distance and the within-class distance of the different rolling bearing fault feature extraction methods are shown in Table 3 and Table 4, respectively. The ratio of the between-class distance to the within-class distance is shown in Table 5. To some extent, the between-class distance and the within-class distance can represent the clustering effect of the feature extraction method. Compared with other feature extraction methods, the fault features extracted by the IMAAPE can have a relatively larger between-class distance and a smaller within-class distance. Although the ratio of the between-class distance to the within-class distance of the IMAAPE is not the best in these methods, its comprehensive performance is the best. Therefore, the fault features extracted by the IMAAPE have good clustering characteristics. The time required by the different feature extraction methods is shown in Table 6. It can be seen from Table 6 that the IMAAPE fault feature extraction algorithm proposed in this paper has higher computational efficiency.

#### 4.1.3. Fault Identification

In order to illustrate the fault identification accuracy of the fault diagnosis method based on the IMAAPE and the mRVM proposed in this paper, the samples under different loads were used to verify the effectiveness of the proposed method. The selection of the experimental samples is shown in Table 1 and Table 2. The experimental results of fault identification accuracy of the different classifiers with different loads are as shown in Table 7, Table 8, Table 9 and Table 10. The fault feature extraction method based on the IMAAPE combined with different classifiers has high fault identification accuracy. Moreover, the experimental results show that the identification accuracy of the mRVM_1__conv_1_ is higher than other classification methods, and at the same time, this method has reasonable operation efficiency. Therefore, the mRVM_1__conv_1_ was used as the multiple classifier of the rolling bearing fault diagnosis method in this paper.

The fault identification accuracy of mRVM_1__conv_1_ under different fault severity with the load 0 hp is shown in Table 11. It can be seen that the fault diagnosis method proposed in this paper can effectively identify the different fault severity of the rolling bearing. Further, the identification accuracy reaches 99.25%. As the selection of nuclear parameters has a great impact on the identification accuracy of the mRVM, this paper compares the selection of nuclear parameters of the mRVM and the experimental results are shown in Table 12. It can be seen that when the nuclear parameter is 8.5, the mRVM multi-classifier has the highest fault identification accuracy for the rolling bearing faults with different fault severity.

In this paper, the effectiveness of the different fault extraction methods combined with the mRVM classifier for the rolling bearing were compared. The experimental results are shown in Table 13. The fault diagnosis method proposed in this paper has the highest fault identification accuracy up to 99.925%.

### 4.2. Fault Diagnosis Experiment of Gearbox

#### 4.2.1. Experimental Platform and Data

**Figure 13:**
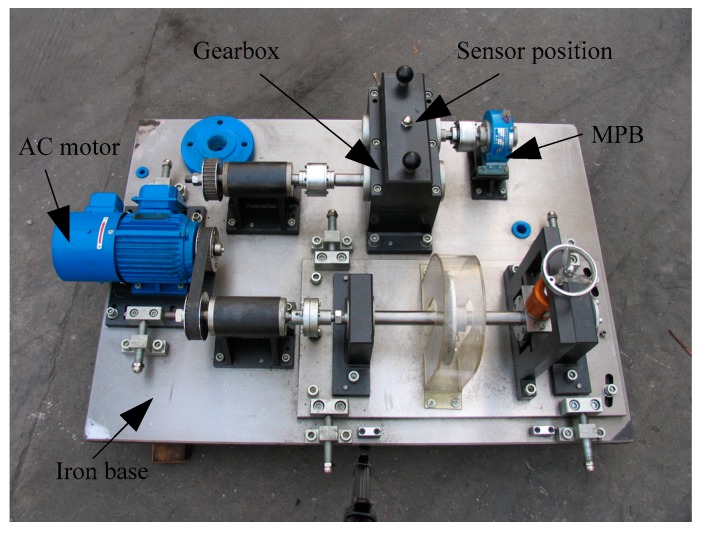
The experimental platform of the gearbox from QPZZ-II.

The experimental platform QPZZ-II was manufactured by Jiangsu Qingpeng Diagnosis Engineering Co., Ltd. A picture of QPZZ-II is shown in Figure 13. The vibration signals were collected by accelerometers. In the experiments, five working states were considered including the normal condition, gear pitting fault (pitting), gear tooth breaking (tooth breaking), pinion wear fault (wearing) and gear pitting fault coupling with pinion wear fault (pitting and wearing). The experimental sample description of the gearbox is shown in Table 14. The acquisition equipment is QPZZ-II produced by the Jiangsu Qianpeng Diagnosis Engineering Co. Ltd. and the sampling frequency is 5.12 kHz. There are 53,248 data points for each health condition. The collected data was divided into several non-overlapping samples. Each sample contained 1024 points.

**Figure 14:**
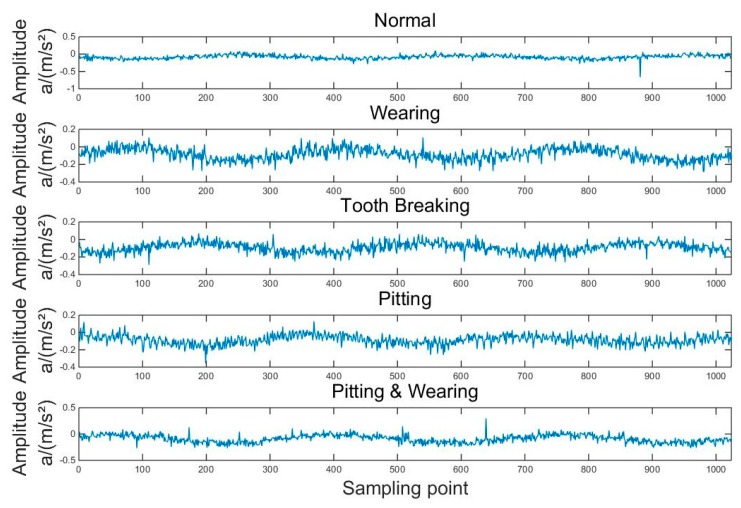
The vibration signal waveforms of the gearbox in different fault conditions.

The vibration signal waveforms of the gearbox in different fault conditions are shown in Figure 14. As can be seen from the figure, the vibration signals of the gearbox under different fault states have differences in amplitude and frequency. This can provide more effective help for the pattern recognition method.

#### 4.2.2. Fault Feature Extraction

**Figure 15:**
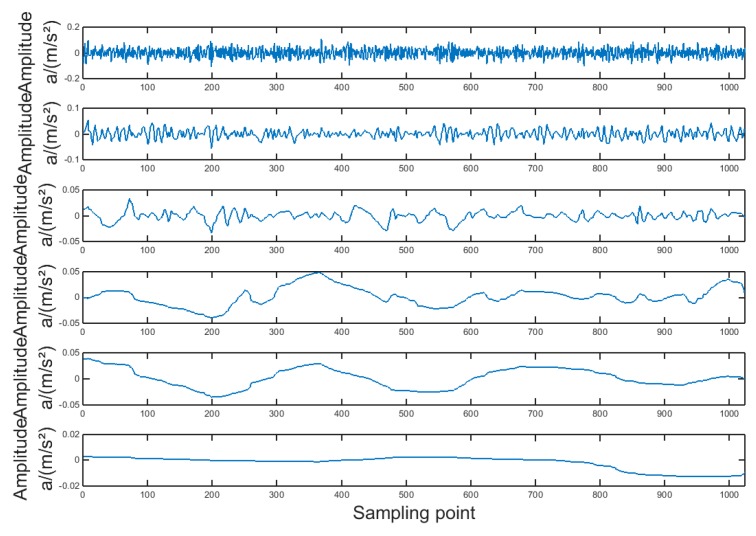
The ITD decomposition results of the vibration signal with the gear pitting fault.

The vibration signal is decomposed by the ITD and the ITD decomposition results (PR components) are shown in Figure 15. The optimum PR component highlights the frequency and amplitude characteristics of the gear pitting fault.

**Figure 16:**
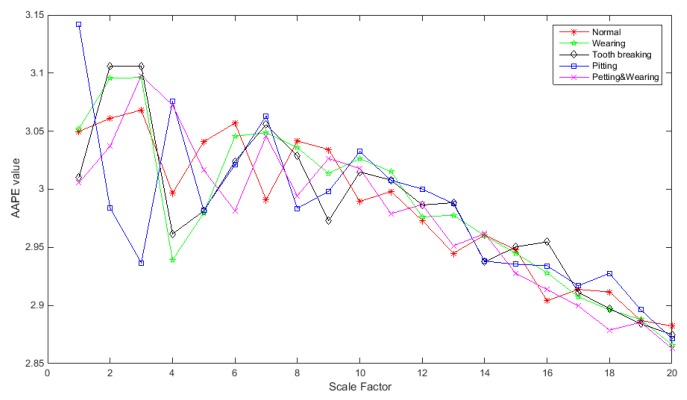
IMAAPE feature vectors for different fault types of the gearbox.

**Figure 17:**
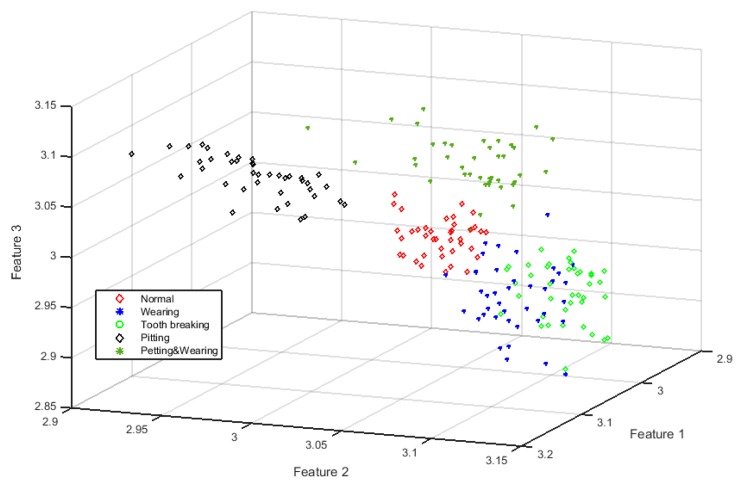
Three dimensions of the IMAAPE feature vectors for different fault types of the gearbox.

Figure 16 shows the IMAAPE feature vectors for different fault types of the gearbox. The IMAAPE can highlight the fault characteristics of the gearbox under different scale factors. The three dimensions of the IMAAPE feature vectors for different fault types of the gearbox are shown in Figure 17. It can be seen from the figure that the IMAAPE fault feature extraction algorithm can effectively describe the fault features and the feature vectors have good clustering characteristics.

#### 4.2.3. Fault Identification

As shown in Table 15, Table 16, Table 17 and Table 18, the selection of nuclear parameters of different mRVM implementations can make the fault identification accuracy of the gearbox up to 100%. In order to evaluate the effectiveness of the gearbox fault diagnosis method proposed in this paper, fault diagnosis accuracy comparison experiments were carried out. The experimental results show that the fault identification accuracy of the IMAAPE and the mRVM based fault diagnosis methods reach 100% in Table 19. Compared with other fault feature extraction methods, the IMAAPE has obvious advantages in fault identification accuracy. Therefore, the proposed fault diagnosis method proposed in this is suitable for gearbox fault diagnosis and has high identification accuracy.

## 5. Conclusions

This paper presents a novel diagnosis method for rotating machinery, which can further analyze the fault severity of rotating machinery on the basis of accurately identify the fault types. The experiments were conducted to illustrate the validity and feasibility of the fault diagnosis method for rotating machinery. This paper can summarize the following conclusions:

(1) The improved multiscale amplitude-aware permutation entropy (IMAAPE) proposed in this paper improves the coarse-graining process of the MSE and the problems existing in the PE, and can effectively extract the fault information contained in the vibration signals. Moreover, compared with other fault feature extraction methods, the IMAAPE has higher execution efficiency.

(2) The multiclass relevance vector machine (mRVM) is suitable for the multi-classification of rotating machinery and has high identification accuracy on the basis of reasonable selection of nuclear parameters.

(3) The rolling bearing experiments and gearbox experiments show the effectiveness of the proposed method. The experimental results on the rolling bearing and gear box show that the proposed fault diagnosis method for rotating machinery has a high fault identification accuracy of over 99%. In particular, the rolling bearing experiments show the potential application of the proposed method in fault severity analysis.

## Figures and Tables

**Table 1:** Experimental sample description of the rolling bearing.

Fault Type	Labels	Fault Diameter (mils)	Training Sample Number	Testing Sample Number
Normal	Norm	0	10	40
Inner race fault	IR07	7	10	40
	IR14	14	10	40
	IR21	21	10	40
Outer race fault	OR07	7	10	40
	OR14	14	10	40
	OR21	21	10	40
Ball elements fault	BE07	7	10	40
	BE14	14	10	40
	BE21	21	10	40

**Table 2:** Experimental sample distribution under different load conditions of the rolling bearing.

Labels	Fault Severity	Fault Diameter (mils)	Load (hp)			
			0	1	2	3
Norm	—	0	✓^1^	✓	✓	✓
IR07	Minor	7	✓	✓	✓	✓
IR14	Medium	14	✓	✓	✓	✓
IR21	Serious	21	✓	✓	✓	✓
OR07	Minor	7	✓	✓	✓	✓
OR14	Medium	14	✓	✓	✓	✓
OR21	Serious	21	✓	✓	✓	✓
BE07	Minor	7	✓	✓	✓	✓
BE14	Medium	14	✓	✓	✓	✓
BE21	Serious	21	✓	✓	✓	✓

^1^ “✓” represents the experimental sample of rolling bearing under current load.

**Table 3:** The between-class distance of different rolling bearing fault feature extraction methods.

Between-Class Distance	IMAAPE	IMPE	RCMPE	IMSE	IMFE	RCMSE
BE-IR	1.373	1.011	1.008	0.933	0.777	0.477
BE-Norm	1.115	1.129	1.159	1.821	5.093	5.693
BE-OR	1.012	0.536	0.534	3.334	2.425	2.293
IR-Norm	1.131	1.111	1.132	1.550	4.982	5.778
IR-OR	1.407	0.808	0.804	3.155	2.320	2.374
Norm-OR	1.385	1.117	1.141	2.554	6.745	7.495

**Table 4:** The within-class distance of different rolling bearing fault feature extraction methods.

Within-Class Distance	IMAAPE	IMPE	RCMPE	IMSE	IMFE	RCMSE
BE	0.183	0.189	0.306	1.087	0.269	0.252
IR	0.187	0.189	0.213	0.944	0.219	0.156
Norm	0.311	0.356	0.450	1.044	0.561	1.014
OR	0.135	0.176	0.233	0.691	0.280	0.105

**Table 5:** The ratio of the between-class distance to the within-class distance.

Failure States	IMAAPE	IMPE	RCMPE	IMSE	IMFE	RCMSE
BE	6.375	4.720	2.942	1.867	10.279	11.194
IR	6.971	5.168	4.607	1.991	12.297	18.438
Norm	3.892	3.143	2.542	1.892	9.994	6.235
OR	9.393	4.661	3.546	4.362	13.679	38.610

**Table 6:** Time required by different feature extraction methods.

Feature Extraction Method	IMAAPE	IMPE	RCMPE	IMSE	IMFE	RCMSE
Time (ms)	0.39	0.57	1.11	0.42	17.30	0.40

**Table 7:** Fault identification accuracy of different classifiers with the load 0 hp.

Classifier Type	Average Time (ms)	Identification Accuracy (%)		
		Maximum	Minimum	Average
mRVM_1__conv_1_	0.3287	100	99.25	99.925
mRVM_1__conv_2_	0.3298	100	97.00	99.842
mRVM_2__conv_A_	0.4465	99.75	97.50	99.05
mRVM_2__conv_N_	9.1430	100	96.75	98.89
OAO-SVM	0.0054	89.00	89.75	89.25
OVA-SVM	0.0241	89.50	89.75	89.53
Random Forest	3.0870	89.25	88.75	88.78

**Table 8:** Fault identification accuracy of different classifiers with the load 1 hp.

Classifier Type	Average Time (ms)	Identification Accuracy (%)		
		Maximum	Minimum	Average
mRVM_1__conv_1_	0.2801	100	100	100
mRVM_1__conv_2_	0.3174	100	100	100
mRVM_2__conv_A_	0.3584	100	98.99	98.73
mRVM_2__conv_N_	10.3659	100	98.74	98.52
OAO-SVM	0.0039	95.00	88.94	92.76
OVA-SVM	0.0241	95.98	93.72	95.18
Random Forest	2.9706	99.00	97.75	98.42

**Table 9:** Fault identification accuracy of different classifiers with the load 2 hp.

Classifier Type	Average Time (ms)	Identification Accuracy (%)		
		Maximum	Minimum	Average
mRVM_1__conv_1_	0.3107	100	100	100
mRVM_1__conv_2_	0.2809	100	100	100
mRVM_2__conv_A_	0.3584	100	100	100
mRVM_2__conv_N_	11.4355	100	100	100
OAO-SVM	0.0039	98.75	87.25	93.00
OVA-SVM	0.0241	99.25	83.25	91.54
Random Forest	2.9706	99.50	99.25	99.40

**Table 10:** The fault identification accuracy of different classifiers with the load 3 hp.

Classifier Type	Average Time (ms)	Identification Accuracy (%)		
		Maximum	Minimum	Average
mRVM_1__conv_1_	0.2855	100	100	100
mRVM_1__conv_2_	0.2662	100	100	100
mRVM_2__conv_A_	0.3584	99.50	98.50	99.30
mRVM_2__conv_N_	11.4355	100	98.75	99.62
OAO-SVM	0.0052	89.72	84.46	86.81
OVA-SVM	0.0222	99.25	83.25	91.54
Random Forest	2.8600	99.75	98.99	99.30

**Table 11:** Fault identification accuracy of the mRVM_1__conv_1_ under different fault severity with the load 0 hp (%).

Testing Fault Severity	Testing Results									
	BE07	BE14	BE21	IR07	IR14	IR21	Norm	OR07	OR14	OR21
BE07	100									
BE14		100								
BE21			100							
IR07				99.25	0.75					
IR14					100					
IR21						100				
Norm							100			
OR07								100		
OR14									100	
OR21										100

**Table 12:** The influence of nuclear parameter selection of the mRVM on fault identification accuracy of the rolling bearing (%).

Testing Fault Severity	Nuclear Parameter Value																	
	1.5	2.0	2.5	3.0	3.5	4.0	4.5	5.0	5.5	6.0	6.5	7.0	7.5	8.0	8.5	9.0	9.5	10.0
BE07	76.25	84.25	91.0	92.25	90.5	93.25	95.5	98.75	100	99.25	93.0	100	100	96.75	100	100	99.50	100
BE14	75.25	87.5	100	98.3	100	93.75	97.0	96.25	100	100	100	99.75	100	100	100	100	99.75	96.50
BE21	59.0	84.5	86.25	92.75	98.75	96.5	99.5	100	99.75	100	99.75	100	100	100	100	100	100	100
OR07	77.0	84.5	88.75	91.75	92.75	98.25	100	99.5	100	100	99.75	100	100	100	100	100	100	100
OR14	75.25	76.0	86.75	100	100	94.0	95.5	97.75	100	100	100	100	100	100	100	95.25	100	100
OR21	76.50	82.0	86.25	84.75	100	97.0	100	100	100	100	100	99.75	99.75	100	100	100	100	97.0
IR07	75.25	84.25	87.0	91.5	92.25	100	94.25	98.25	100	100	100	100	100	100	99.25	95.25	100	100
IR14	76.50	82.75	90.0	89.25	88.5	93.75	96.75	99.75	99.25	100	100	100	100	100	100	100	100	100
IR21	59.0	84.5	86.25	92.75	98.0	96.0	99.25	97.75	98.75	99.0	97.5	100	100	100	100	100	100	97.0
Norm	75.0	84.75	93.25	100	94.25	100	90.75	97.0	100	99.0	100	100	100	100	100	100	100	100

**Table 13:** Rolling bearing fault identification accuracy of different fault extraction methods combined with the mRVM.

Feature Extraction Method	Fault Identification Accuracy (%)
IMAAPE	99.925
IMFE	96.25
IMPE	96.0
RCMSE	92.25
IMSE	84.25
RCMPE	97.5

**Table 14:** Experimental sample description of the gearbox.

Fault Type	Labels	Training Sample Number	Testing Sample Number
Normal	1	10	40
Wearing	2	10	40
Tooth breaking	3	10	40
Pitting	4	10	40
Pitting & wearing	5	10	40

**Table 15:** The influence of nuclear parameter selection of the mRVM_1__conv_1_ on fault identification accuracy of the rolling bearing (%).

Testing Fault Severity	Nuclear Parameter Value																	
	1.5	2.0	2.5	3.0	3.5	4.0	4.5	5.0	5.5	6.0	6.5	7.0	7.5	8.0	8.5	9.0	9.5	10.0
BE07	92.0	100	98.5	100	99.5	100	100	100	100	100	100	100	100	100	100	100	100	100
BE14	77.5	92.0	99.0	97.0	100	100	100	100	100	100	100	100	100	100	100	100	100	100
BE21	91.0	97.0	99.5	100	100	100	100	100	100	100	100	100	100	100	100	100	100	100
OR07	96.5	99.0	100	98.5	99.0	100	100	100	100	100	100	100	100	100	100	100	100	100
OR14	93.0	97.5	99.0	100	99.0	100	100	100	100	100	100	100	100	100	100	100	100	100
OR21	63.0	94.0	99.0	100	99.0	100	100	100	100	100	100	100	100	100	100	100	100	100
IR07	90.0	100	100	99.5	99.0	100	100	100	100	100	100	100	100	100	100	100	100	100
IR14	63.0	97.5	100	100	100	100	100	100	100	100	100	100	100	100	100	100	100	100
IR21	95.0	99.0	98.5	99.0	100	100	100	99.5	100	100	100	100	100	100	100	100	100	100
Norm	98.5	98.5	91.0	100	100	100	100	100	100	100	100	100	100	100	100	100	100	100

**Table 16:** The influence of nuclear parameter selection of the mRVM_1__conv_2_ on fault identification accuracy of the rolling bearing (%).

Testing Fault Severity	Nuclear Parameter Value																	
	1.5	2.0	2.5	3.0	3.5	4.0	4.5	5.0	5.5	6.0	6.5	7.0	7.5	8.0	8.5	9.0	9.5	10.0
BE07	78.5	93.0	100	100	100	100	100	100	100	100	100	100	100	100	100	100	100	100
BE14	96.5	100	100	100	98.5	100	100	100	100	100	100	100	100	100	100	100	100	100
BE21	92.0	100	91.0	100	100	100	100	100	100	100	100	100	100	100	100	100	100	100
OR07	99.0	100	99.0	100	100	100	100	99.5	100	100	100	100	100	100	100	100	100	100
OR14	96.5	91.0	99.5	100	100	99.0	100	100	100	100	100	100	100	100	100	100	100	100
OR21	95.0	78.0	100	99.0	100	100	100	100	100	100	100	100	100	100	100	100	100	100
IR07	70.0	100	99.0	95.0	98.5	100	100	100	100	100	100	100	100	100	100	100	100	100
IR14	80.0	100	94.0	98.5	100	100	100	100	100	100	100	100	100	100	100	100	100	100
IR21	99.0	96.0	99.5	100	100	100	100	99.5	100	100	100	100	100	100	100	100	100	100
Norm	85.0	97.0	99.5	100	100	100	100	100	100	100	100	100	100	100	100	100	100	100

**Table 17:** The influence of nuclear parameter selection of the mRVM_2__conv_A_ on fault identification accuracy of the rolling bearing (%).

Testing Fault Severity	Nuclear Parameter Value																	
	1.5	2.0	2.5	3.0	3.5	4.0	4.5	5.0	5.5	6.0	6.5	7.0	7.5	8.0	8.5	9.0	9.5	10.0
BE07	100	100	100	100	100	100	100	100	100	100	100	100	100	100	100	100	100	100
BE14	98.0	100	99.5	100	100	100	100	100	100	100	100	100	100	100	100	100	100	100
BE21	94.0	100	100	100	99.5	100	100	100	100	100	100	100	100	100	100	100	100	100
OR07	100	100	100	100	100	100	100	99.5	100	100	100	100	100	100	100	100	100	100
OR14	100	97.5	100	100	100	99.0	100	100	100	100	100	100	100	100	100	100	100	100
OR21	99.5	100	96.5	100	100	100	100	100	100	100	100	100	100	100	100	100	100	100
IR07	100	100	100	100	100	100	100	100	100	100	100	100	100	100	100	100	100	100
IR14	100	100	100	100	100	100	100	100	100	100	100	100	100	100	100	100	100	100
IR21	97.5	96.0	99.5	100	100	100	100	100	100	100	100	100	100	100	100	100	100	100
Norm	87.0	99.5	100	100	100	100	100	100	100	100	100	100	100	100	100	100	100	100

**Table 18:** The influence of nuclear parameter selection of the mRVM_2__conv_N_ on fault identification accuracy of the rolling bearing (%).

Testing Fault Severity	Nuclear Parameter Value																	
	1.5	2.0	2.5	3.0	3.5	4.0	4.5	5.0	5.5	6.0	6.5	7.0	7.5	8.0	8.5	9.0	9.5	10.0
BE07	70.5	81.5	85.5	88.5	96.5	97.5	98.5	100	99.0	99.5	99.5	99.5	99.5	100	100	100	100	100
BE14	84.5	82.0	82.5	89.0	91.5	97.5	98.0	100	100	99.5	99.5	99.5	100	100	100	100	100	100
BE21	79.0	81.5	85.5	90.5	94.0	96.0	97.5	99.0	99.5	99.5	100	100	100	100	100	100	100	100
OR07	78.0	83.5	87.0	91.0	95.0	97.5	98.5	99.0	99.0	100	99.5	99.5	99.5	100	100	100	100	100
OR14	79.5	79.0	86.5	86.0	92.5	97.0	97.5	100	99.5	100	100	100	100	100	100	100	100	100
OR21	78.0	84.0	81.0	91.5	97.0	99.0	96.5	99.5	99.5	99.5	100	99.5	100	100	100	100	100	100
IR07	77.0	80.5	86.0	91.5	94.0	96.5	97.5	98.5	100	100	99.5	99.5	100	100	100	100	100	100
IR14	73.5	79.0	85.5	88.5	94.5	97.5	100	99.5	100	100	99.5	99.5	100	100	100	100	100	100
IR21	81.0	77.5	85.0	87.5	96.0	97.0	98.5	100	99.5	100	100	100	100	100	100	100	100	100
Norm	67.0	78.5	86.5	90.0	95.5	98.0	96.5	99.5	100	99.5	100	100	100	100	100	100	100	100

**Table 19:** Gearbox fault identification accuracy of different fault extraction methods combined with the mRVM.

Feature Extraction Method	Fault Identification Accuracy (%)
IMAAPE	100
IMFE	99.0
IMPE	98.0
RCMSE	99.5
IMSE	96.5
RCMPE	97.5

## Abbreviations

AAPE	Amplitude-aware permutation entropy
ApEn	Approximate entropy
ANN	Artificial neural network
BE	Ball elements
CEEMD	Complementary ensemble empirical mode decomposition
EMD	Empirical mode decomposition
ELCD	Ensemble local characteristic-scale decomposition
FFT	Fast Fourier transform
IMAAPE	Improved multiscale amplitude-aware permutation entropy
ISVM-BT	Improved support vector machine based on binary tree
IR	Inner race
IMF	Intrinsic mode function
IMFE	Improved multiscale fuzzy entropy
IMSE	Improved multiscale entropy
ISC	Intrinsic scale component
ITD	Intrinsic time-scale decomposition
LMD	Local mean decomposition
mRVM	Multiclass relevance vector machine
MSE	Multiscale entropy
MFE	Multiscale fuzzy entropy
MPE	Multiscale permutation entropy
Norm	Normal
OVA-SVM	One-against-All SVM
OAO-SVM	One-against-One SVM
OR	Outer race
PE	Permutation entropy
PNN	Probabilistic neural network
PF	Product function
PR	Proper rotation
RF	Random forest
RCMPE	Refined composite multiscale permutation entropy
RCMSE	Refined composite multiscale entropy
RVM	Relevance vector machine
SampEn	Sample entropy
SVM	Support vector machine
TFE	Time-frequency-energy
WPT	Wavelet packet transform
WT	Wavelet transform
